# Editorial note

**DOI:** 10.1098/rsta.2022.0093

**Published:** 2022-08-22

**Authors:** Editorial office

**Affiliations:** Transactions A; Royal Society

This issue is the second part of a two-part issue on ‘The future of mathematical cosmology’. Volume 1 can be found at https://royalsocietypublishing.org/toc/rsta/2022/380/2222. The Preface to Volume 1 contains a brief summary of the topic for both issues [1].
